# Epidemiology and immunoprotection of nephropathogenic avian infectious bronchitis virus in southern China

**DOI:** 10.1186/1743-422X-8-484

**Published:** 2011-10-27

**Authors:** Qingmei Xie, Jun Ji, Jingwei Xie, Feng Chen, Manshan Cai, Baoli Sun, Chunyi Xue, Jingyun Ma, Yingzuo Bi

**Affiliations:** 1 College of Animal Science, South China Agricultural University, Guangzhou 510642, China; 2 Guangdong Wen's Foodstuffs Group Co. Ltd., Yunfu 527439, China; 3 State Key Laboratory of Livestock and Poultry Breeding, Institute of Animal Science, Guangdong Academy of Agricultural Sciences, Guangzhou 510640, China; 4 State Key Laboratory of Biocontrol, College of Life Sciences, Sun Yat-Sen University, Guangzhou 510006, China

## Abstract

**Background:**

In last three years, 96 suspected poultry farms from different provinces in China were diagnosed for avian infectious bronchitis (IB) survey. Finally, 221 IBV strains were confirmed by dwarf embryo test and RT-PCR assay. By virus recovery trials, 187 of the isolates caused the birds died or distressed from nephritis, which was accordant with the clinical record.

**Results:**

Based on epidemiology analysis of recent field isolates of nephropathogenic IB in vaccinated farms in China, YL6 strain were used for vaccination and evaluated by antibody titer and challenge tests. The immunoprotection test indicated that the practical application of vaccine based on the recent field strains could finely facilitate controlling the nephropathogenic IB.

**Conclusions:**

Our study was aim at setting a guide for safeguard against nephropathogenic IBV-caused disease in China.

## Background

Infectious bronchitis (IB) is a serious and highly contagious disease of chickens all over the world. Avian infectious bronchitis virus (IBV) was first reported in the USA for replicating in the respiratory tract and some other epithelial cells of gut, kidney, and oviduct. Subsequently, some strains of IBV caused pathology in non-respiratory organs (such as kidney and gonads) were documented [1]. The clinical disease and production problems frequently cause catastrophic economic losses to the poultry industry, accompanied by decreased production performance in breeder flocks, diminished egg production and poor egg quality in laying flocks [1-3]. In China, IB has a more profound social impact for chicken industrial contributes to the rural economy. More importantly, there is accumulating evidence that nephropathogenic type IB has been more and more prevalent in China recently, but the strains isolated in earlier years mainly caused respiratory signs, which suggested that selecting and immunization with the appropriate vaccine strain is of great importance to control IB infection [4-7].

Some researchers reported that satisfactory cross protection could be provided by appropriate vaccine programs against genetically or antigenically unrelated IBVs [8]. However, this symphysial vaccine manner was restricted by the diversity of the IBV strains. Since IBV strains were first isolated and identified in China in 1982, various live-attenuated and inactivated vaccines derived from respiratory-typed strains have been widely and extensively used in chicken farms to reduce the adverse effect of the IBV [6,7]. However, the disease continues to emerge and cause serious production problems, even occurred in routinely vaccinated layer and breeder flocks in China, and the situation gets worse as time progressed [9]. The most possible explanation for this phenomenon may be that the vaccine effectiveness is diminished by poor cross-protection against the circulating strains.

The spike tip glycoprotein (S1) of virus particle has direct relation to induce virus neutralizing antibody, and determines the cross-protection [10,11]. Our previous research had confirmed that the predominant IBVs were nephropathogenic IBVs were mainly A2-like strains in China during 2008-2009 [4]. This study was to further investigate the prevalence of nephropathogenic IB under immune pressure with routine vaccine strains in China. Additionally, the effectiveness of vaccination program using the common field strains practically against IB was also verified.

## Results

### Isolated IBV strains during 2008 to 2010

From unhealthy birds suspected of IBV infection in the vaccinated chicken farms from Guangdong, Guangxi, Fujian, Hainan, Jiangsu, Zhejiang, Chongqing, Hubei, Sichuan and Jiangxi province of China, 221 filed IBV strains isolated during 2008-2010 were confirmed by RT-PCR, including 214 strains isolated from broilers, and 7 strains from broiler breeder. The isolation rates in the three years were season-dependent to some extent, 58 strains were isolated in early summer (April and May) and 99 strains were isolated in fall and winter (from September to January). The ages of flocks at the time of the outbreak varied between 4 and 70 days. Nearly half (110/221) of the isolated strains were isolated from the chickens between 10 to 30 days of age, seven strains below 10 days, and six strains above 60 days. Eighty of the 221 isolates have been molecular analyzized to be mainly A2-like strains [4].

Since the fourth day post-inoculation, most of the chicks were listless and huddled together, showed appetite and weight lose. The virus recovery tests indicated 84.6% (187/221) isolates caused serious kidney lesions, which were presented with swollen specked kidney and distended ureters filled with uric acid, and the other isolates caused respiratory system signs. All the above were consistent with the clinical record of each strain.

### Vaccination test and challenge protection

After the birds were vaccinated, sera were collected and identified by ELISA, the antibody titer was kept ascending slowly, on 10 days after the second immunization reached to a considerable level, even better before challenge, but none in the control group 4 (unvaccinated but challenged).

As showed in Table 1, there wasn't obvious discrepancy in the antibody levels of Group 1, 2 and 3, inoculated with vaccine XZB and YL6, but much higher than the control Group 4. The chicks at 24 days old of Group 3 were a bit higher than Group 1 and 2. Since the second day post challenge with IBVs, all of the chickens in the four groups showed transitory and mild respiratory signs, including tracheal rales, sneezing and coughing. Postmortem examination of dead and sacrificed birds, revealed the renal petechial haemorrhages, urates deposition and degenerative changes in renal tubules. While in the chickens of group 3, there was no morbidity, and all of the clinical signs of these challenged birds tended to disappear gradually after two weeks of challenge test (Table 2). Finally, the birds were validated to be no virus detected by virus isolation through tracheal swabs after three weeks post challenge. The results of vaccine-challenge trials indicated that combined immunization with vaccine XZB and YL6 used in chickens could induce prevention against the infection of nephropathogenic IBV. The protection effect of XZB (vaccinated once or twice) was weaker.

**Table 1:** Antibody titers before challenge (Mean value)

Group	5 days old	10 days old	17 days old	24 days old
1	2.32 ± 1.242	21.73 ± 7.406	364.41 ± 37.253^a^	851.34 ± 58.734^a^
2	2.32 ± 1.221	22.75 ± 7.306	477.76 ± 31.132^a^	1316.26 ± 87.820^b^
3	2.12 ± 1.226	19.69 ± 6.121	471.73 ± 4.436^a^	1612.38 ± 69.443^b^
4	2.11 ± 1.222	2.63 ± 8.481	2.12 ± 2.855^b^	2.23 ± 1.212^c^

^a, b, c ^The difference between data with the same letters in the same column is not significant (P > 0.05). The difference between data with different letters in the same column is significant (P < 0.05).

**Table 2:** Challenge-vaccination trial program

Group	1^st ^Vaccination (5 days old)	1^st ^Vaccination Dose	2^nd ^Vaccination (10 days old)	2^st ^Vaccination Dose	Death rate (%)
1	H120+W93	0.03 ml^1^	PBS	0.2 ml	35
2	H120+W93	0.03 ml	H120+W93	0.03 ml	15
3	H120+W93	0.03 ml	YL6	0.2 ml	0
4	PBS	0.03 ml	PBS	0.2 ml	100

^1 ^Dose per chicken, consist of H120 (10^3.5^EID_50_) and W93 (10^4.7^EID_50_) at least.

After five-month practical application in the field, it was proved that using the YL6 inactivated vaccine could basically control the prevalence of nephropathogenic IB, inducing excellent protection (94%). The broilers and breeders would not be infected, and the satisfactory immunity would prevent the hens from lower fertilized rate, reduced egg quality and laying quantity, and the progeny chicken could get a high level of maternally antibodies.

## Discussion

Infectious bronchitis (IB) is one of the most common and difficult-control poultry diseases in China, caused persistent but infrequent outbreaks in commercial chicken farms [9,12,13]. The clinical cases caused by vaccination failure raised the importance to investigate the circulating nephropathogenic IBVs and select the candidate vaccine strain against the infections [1,12].

In this study, 221 IBV strains were isolated from the vaccinated chicken flocks, with a wide age range of IB outbreak, 51.6% (117/221) of the birds were not older than 30 days. The chickens infected before the age of 10 days which might be resulted from the maternal antibody could not provide pertinent protection against the prevalent strains. Nowadays, various nephropathogenic strains of IBV have been identified throughout the world. The prevalence of nephropathogenic IB becomes to be a problem for chicken industry in China in recent few years [4,5,7]. Through clinical records and the virus recovery trials, 187 identified isolates mainly caused typical swollen kidney, different from the respiratory type strains isolated in earlier years, including the major vaccine strains.

The vaccine-challenge tests further demonstrated that chicks vaccinated with currently used vaccines in China cannot provide sufficient protection against the prevalent virus strain. The antibody titers evaluated by ELISA were closely associated with the antigen differences. The complex antigen variations of IB caused the antibody titers were just referenced, which showed no obvious discrepancy between commercial and field vaccine in this study. The effective of vaccines were dependent on the protection rate. Absorbingly, the commercial vaccine associated with the inactivated oil-emulsion vaccine (novel field strains) can satisfactorily prevent the vaccinated birds against the infections. The inactivated oil-emulsion vaccine (YL6) associated with commercial vaccines have been used in some flocks, and effectively controlled the supposed IB outburst from 2009 to 2010.

The S1 protein determined the serotypic evolution, the phenotype change and the genetic diversity of IBVs [11,14]. The commercial vaccine was consisted of H120 strain and W93 strain, the latter one was nephropathogenic strain, but attenuated and started for vaccination in 1990s. The error-prone nature of RNA polymerase made the S1 gene could easily generate mutations to bring about new variation strain. Persistent evolutionary variations caused the distant genetic relationship between the vaccine strains and the circulating strains. Our previous report had determined that nephropathogenic IBVs were mainly A2-like strains in China in recent years, showed evolutionarily distant from vaccine strains [4].

## Conclusions

In conclusion, the data obtained from our study suggested the satisfactory immunoprotection of the YL6 vaccine, which genetically belonged to the A2-type group, used in the southern China. To control the prevalence and well prepare for the potential outbreaks of IB, the candidate virus strain for vaccination might be selected timely. Therefore, continuing surveillance of new IBV strains and selecting the representative virus strain for vaccination would be the most effective manner to reduce the economic losses caused by IB. We hope the study could contribute to guiding the development of effective vaccines and establishment of control policy for nephropathogenic IB.

## Materials and methods

### Virus isolation and identification

During the period from June 2008 to November 2010, IBV surveys were performed in 96 suspected but vaccinated flocks from eastern, southern, southwestern and central China. Documented clinical signs of the birds included respiratory and typical nephropathogenic symptoms. Viruses in the homogenized tissue pool of kidney and trachea of the field isolates were propagated by inoculating via the allantoic cavity of 10 day old SPF embryonated eggs for more than three passages. The embryos dying within 24 hours of inoculation were screened to be nonspecific deaths. The isolates caused stunting, dwarfing, curling of the embryo or the presence of urates in the mesonephros after 5 days incubation were indicative of IB virus replication. The viruses were further confirmed by RT-PCR assays. The allantoic fluids containing IBV isolates after 72 h post inoculation were harvested and preserved in liquid nitrogen.

Total RNA extraction of the allantoic fluid was completed using RNAiso reagent (TaKaRa Biotechnology, Dalian, China) according to the manufacturers' instructions. Reverse transcription polymerase chain reaction (RT-PCR) was carried out by PrimeScriptTM One-Step RT-PCR Kit in 25 μl reaction volume containing 20 μl of RT-PCR PreMix (reaction buffer, dNTPs, 2 μl of enzyme mix), 2 μl of extracted viral RNA and the specific primers (National standard, GBT23197-2008), one primer pair targeting the M gene (Ms: 5'-CCTAAGAACGGTTGGAAT-3', Mx: 5'-TACTCTCTACAC ACACAC-3') and another pair for the 3' UTR of genome (3's: 5'-GGAAGATAGGCATGTAGCTT-3', 3'x: 5'-CTAACTCTATACTAGCCTAT-3').

After IBV confirmation, virus recovery was performed as previous described [4]. Five 1-day-old SPF White Leghorn chickens were intranasally inoculated with each isolate, respectively. All of the chicks were examined and recorded daily for clinical signs for 20 days post inoculation, the dead birds were necrospied for lesions of respiratory tract or nephritis. Finally, all the survivors were sacrificed and necrospied. All of the animal experiments were conducted in accordance with the guidelines of Guangdong Province on the Review of Welfare and Ethics of Laboratory Animals, and under the protocol (SCAU-AEC-2010-1102) approved by the Animal Ethics Committee of South China Agricultural University.

### Vaccination-challenge of immunity trials and clinical application

Based on clinical records and sequence blast from our previous report, YL6 strain (GenBank accession numbers: GU938393 for S1 gene) isolated from Yulin city of Guangxi province in June 2009 (with high morbidity of 100% and mortality of 79%) was selected for preparation of inactivated oil-emulsion vaccine. Ten-day-old SPF embryonated eggs were inoculated with strain YL6 through five passages to yield a titre of 10^6 ^to 10^7 ^median embryo infectious dose (EID50), which were titrated using the method described by Liu et al (2006a). The harvested viruses were inactivated with 2/1000 formaldehyde for 36 h at 37°C, followed by testing for absence of infectivity by embryo inoculation, adjuvanted with mineral oil in the ratio of 1 to 2 finally.

Eighty 1-day-old SPF White Leghorn chickens were housed in biosecurity isolators under quarantine conditions in Wen's Foodstuff Group Company, and randomly allocated into 4 groups with 20 birds each. The protection effect of live attenuated vaccine XZB (Baoite Biopharmaceutical Corp. Ltd., Qingdao, China; Commercial vaccine, consisted of W93 and H120 strain) and YL6 vaccine were compared through the vaccination-challenge trials. The birds were inoculated intranasally with routine XZB or vaccinated intramuscularly with inactivated vaccine YL6. The detailed vaccination procedures were showed in Table 2. Group 4 was reared as vaccine-negative control.

Blood samples were collected on day 5, 10, 17 and 24. Serum antibody titers of 10 randomly selected chickens from each group were diluted (1: 500) and evaluated with a commercial total antibody ELISA kit (IDEXX Corporation, Westbrook, Maine, USA) as described by Liu et al [15]. Optical density (OD) of each well was read in an ELISA plate reader at 650 nm. Serum-to-positive ratios were calculated, using the formula: SP ratio = (OD sample - OD negative control)/(OD positive control - OD negative control). From these SP ratios, individual serum titres were calculated according to the manufacturer's instructions.

Challenge test was performed in biosafety-level (BSL)-3^+ ^lab after the final antibody test to examine whether or not the novel strain vaccines can provide protective immunity against the relevant infectious viruses. All of the chickens at 24 days old were infected by intranasal instillation of 10^5 ^× EID_50 _of virulent nephropathogenic HY51 strain (GenBank accession numbers: GU938386 for S1 gene) isolated from Heyuan city of Guangdong province (with high morbidity of 100% and mortality of 76%) in May 2009, the strain was belonged to the IBVs of predominant genotype [4]. The chicks were examined daily for signs of infection for 30 days after inoculation. The virus isolation was carried out through tracheal swabs after three weeks post challenge.

Sufficient inactivated oil-emulsion vaccine YL6 was produced as described previously, and applied in some chicken farms in southern China, including Guangdong, Guangxi, Guizhou, Fujian and Sichuan province. In the flocks with epidemic history, the vaccination procedures were as follows, broilers were inoculated intranasally with routine XZB at 5 days of age, and vaccinated intramuscularly with 0.2 ml of inactivated vaccine YL6 at 10 days of age; Broilers were vaccinated intramuscularly with 0.3 ml of YL6 again at 20 days of age. In the non-epidemic flocks, broilers were inoculated with routine XZB at 5 days of age, and immunized with 0.2 ml of vaccine YL6 at 10 days of age.

## Competing interests

The authors declare that they have no competing interests.

## Authors' contributions

QX and JJ carried out most of the experiments and wrote the manuscript, and should be considered as first authors. JX and FC critically revised the manuscript and the experiment design. MC, BS, CX, JM and YB helped with the experiment. All of the authors read and approved the final version of the manuscript.
